# Softening Effect on Fracture Stress of Pure Copper Processed by Asynchronous Foil Rolling

**DOI:** 10.3390/ma12142319

**Published:** 2019-07-20

**Authors:** Jingqi Chen, Xianlei Hu, Xianghua Liu

**Affiliations:** State Key Laboratory of Rolling and Automation, Northeastern University, Shenyang 110819, China

**Keywords:** Cu foils, cold rolling, mechanical properties, softening effect

## Abstract

In order to study the size effect on the mechanical property of micro-scale metal, pure copper strips with thicknesses in the range of 20 µm to 600 µm were obtained through the asynchronous foil rolling technology. Progressive mechanical property tests indicated that the pure copper experiences softening effect at a micro-scale when the thickness is below 80 µm, which is contrary to the traditional work hardening theory. The related mechanisms were analyzed and discussed through the observation of microstructure and fracture morphology. The decrease of fracture stress with the decrease of thickness can be attributed to the decreased interfacial energy and dislocation density, which contributes to the release of the cumulative distortion energy and the tendency to soften. In addition, the distribution of misorientation angle and changed Taylor factor with the decrease of thickness are other important factors. The fracture morphology indicated a reduction in the number of micro-voids and the nature of fracture transformed from dimpled pattern to knife edge rupture with thickness. The traditional Hall-Petch relationship is no longer applicable due to the softening effect. A modified Hall-Petch relation considering the distribution of misorientation angle and Taylor factor was established, which provided a better relationship between flow stress and grain size.

## 1. Introduction

With the rapid development of product miniaturization, the demand on micro-scale products and parts has increased significantly in many industries such as electronics, communications, healthcare, and aerospace [1]. In particular, micro-scale pure copper parts are widely used in electrical products such as battery electrodes in electric cars and flexible printed circuits in cell phones and computers [2,3]. However, when the sample size is reduced from macro-scale to micro-scale, the mechanical properties and fracture behavior of the material may change because of the size effect [4,5]. The traditional understanding and material models are not valid in the micro-forming processes [6,7].

Several studies have been focused on the size effect on micro-forming. Sinclair et al. [8] studied the size effect on work hardening of polycrystalline copper at low temperature. It was found that the dynamic recovery at boundaries at large strain eliminates grain size effect on work hardening. Chen et al. [9] proposed two models to predict the forming limit of stainless steel 304 foils in micro-forming with the consideration of size effect and ductile fracture criterion. Fan [10] established a composite model to describe the size effect on fracture behavior by treating polycrystalline material as a composite consisting of grain interior and grain boundary. Kals and Eckstein [11] compared different size effects on a sheet metal in the experiments of air bending, tensile test, and punching. The fracture surface of tensile specimens showed that localized shearing and substantial necking are more likely to occur when the specimen dimension is miniaturized. Molotnikov et al. [12,13] established a model to describe the size effect on tensile strength with the consideration of the ratio of the sheet thickness to grain size. Furushima et al. [14] found that the traditional ductile fracture criterion is not applicable to predict the fracture behavior of metal foil.

When the sample size in one direction is reduced from macro-scale to micro-scale, one needs to be careful to use the classical Hall-Petch relationship. Some studies focused on the size-effect on the Hall-Petch relationship. Hansen [15] reported that boundary strengthening cannot be regarded as a constant and the Hall-Petch relation must be modified for deformed micro-scale metals. Wang et al. [16] analyzed the size effect on flow stress of pure nickel polycrystals during uniaxial compression test with the consideration of boundary conditions. It was found that an inverse Hall-Petch relation occurs when the ratio of thickness to grain size is less than about 6. Kim et al. [17] studied the sample and grain size effects on the micro-forming process and established a scaling model based on the Hall-Petch relationship, while Song et al. [18] proposed coherent polycrystal model to explain the inverse Hall-Petch relation in nanocrystalline materials.

In this study, the tensile tests on pure copper strips with thicknesses in the range of 20 µm to 600 µm were carried out to study the softening effect on micro-scale plastic deformation. The fracture stress decreased with reduction in thickness and is attributed to the influence of grain size and dislocation density. The distribution of misorientation angle and Taylor factor were observed and measured by electron backscatter diffraction (EBSD) and incorporated into the classical Hall-Petch relationship to explain the relationship between the flow stress and grain size in micro forming. 

## 2. Experimental Procedure

### 2.1. Material 

**Figure 1:**
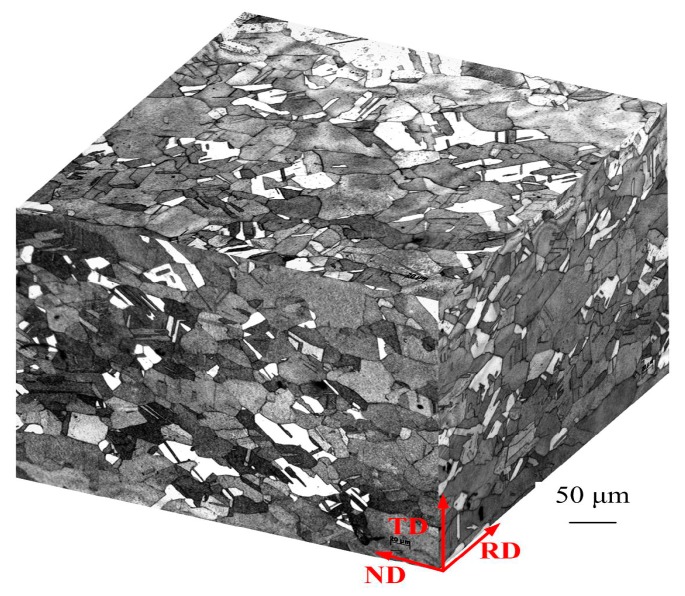
Microstructure of the as-received specimen along the rolling direction (RD), transverse direction (TD), and normal direction (ND).

The as-received copper strips with thicknesses of 5 mm were annealed at 500 °C for 30 min in vacuum condition. The average grain size of the as-received specimen is ~50 µm along the rolling direction (RD), transverse direction (TD), and normal direction (ND). The chemical composition was measured by X-ray fluorescence and is listed in Table 1. The microstructure of the as-received specimen is shown in Figure 1. To obtain different thicknesses, the samples were cold rolled at room temperature without intermediate annealing. 

**Figure 2:**
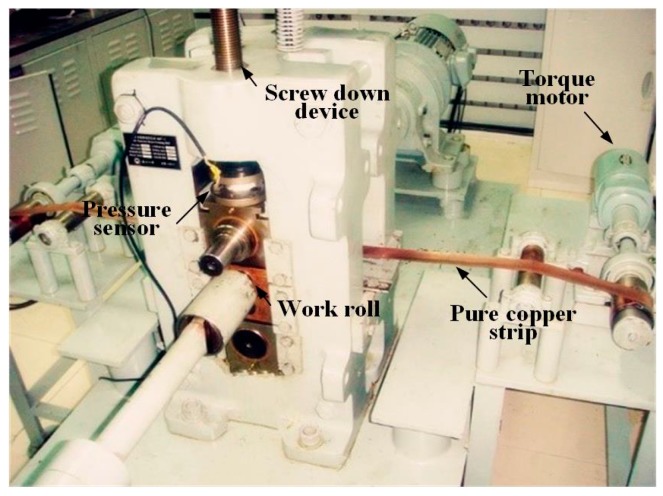
Illustration of 3M mill (micro metal forming mill).

The 3M mill (micro metal forming mill, product model, manufacturer, city, country) was used for the foil rolling, which is a new type of asymmetric rolling mill developed by our group, shown in Figure 2. There is an advantage in 3M mill, in which it can continuously adjust the speed ratio by means of setting the speed for the up and down roll respectively. We rolled from 5 mm to 0.05 mm after 20 passes and the reduction per pass was ~25%. 

### 2.2. Tensile Test 

The tensile samples with a gage length of 25 mm and a width of 10 mm were prepared by electrical discharge machining (Dk77, Musee Home, Shenyang, China). The uniaxial tensile test was carried out using a constant strain rate of 10^−4^ s^−1^ at room temperature. Five tensile samples were tested for each thickness. After the tests, the fracture surfaces of the samples were observed via a scanning electron microscope (SEM, Ultra 55, Zeiss, Oberkochen, Germany). The microstructural examination was carried out by EBSD (Ultra 55, Zeiss, Oberkochen, Germany) and transmission electron microscopy (TEM). Specifically, EBSD measurements were conducted over the area of 10 μm × 14 μm with a step size of 0.05 μm at a voltage of 30 kV. The TEM study was carried out with a Tecnai G220 (FEI, Hillsboro, OR, USA) at 200 kV. 

## 3. Results

### 3.1. Softening Effect on Mechanical Properties

**Figure 3:**
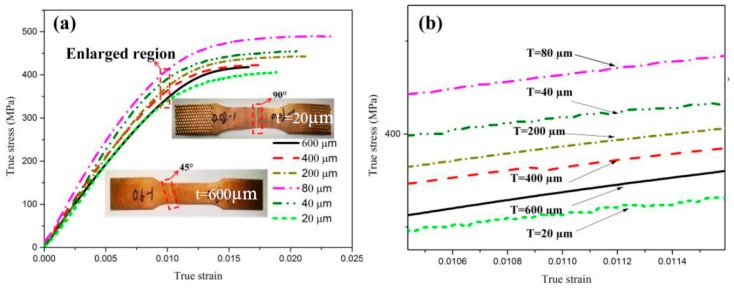
Experimental results. (**a**) Stress–strain curves, (**b**) enlarged region. The fractography of tensile samples with thicknesses of 600 µm and 20 µm after uniaxial tensile test is shown in (**a**).

Figure 3 presents the flow stress–strain curves of the pure copper samples obtained from the uniaxial tensile test. There is no obvious yield phenomenon in the curves for both Cu sheets and foils. In order to clearly observe the stress–strain relationship in Figure 3a, Figure 3b shows the detailed image of the dotted rectangle in Figure 3a. When the Cu sheet was deformed, dislocations were generated and piled-up leading to work hardening. The fracture stress thus increased from 417 MPa to 489 MPa when the thickness of Cu sheet decreased from 600 µm to 80 µm. However, an obvious softening effect [19] was observed when the thickness of rolled foil was less than 80 µm. The fracture stress decreased from 489 MPa to 406 MPa when the thickness was decreased from 80 µm to 20 µm. The thinner foil had a lower fracture stress. The shear stress is of great significance to the fracture process. The fractography of tensile samples after uniaxial tensile test is shown in Figure 3a. It is obvious that the samples with thicknesses of 600, 400, 200 µm fractured ~45° to the vertical axis. However, the fracture was ~90° to the vertical axis with thicknesses of 80, 40, 20 µm.

### 3.2. Fracture Surface

**Figure 4:**
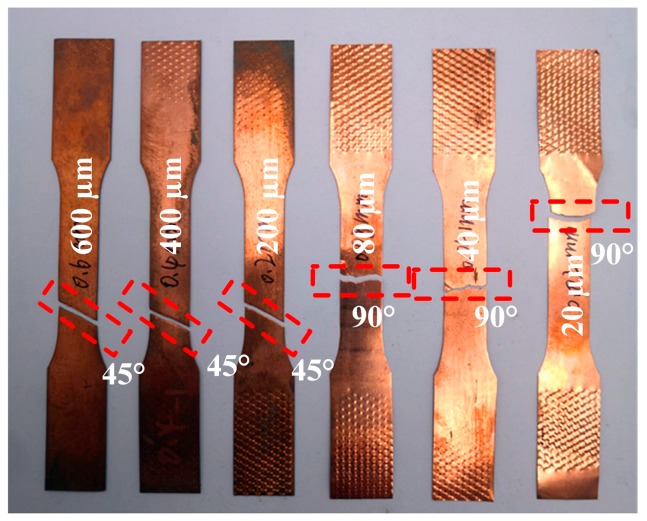
Fracture angle of tensile tested specimens of different thicknesses.

**Figure 5:**
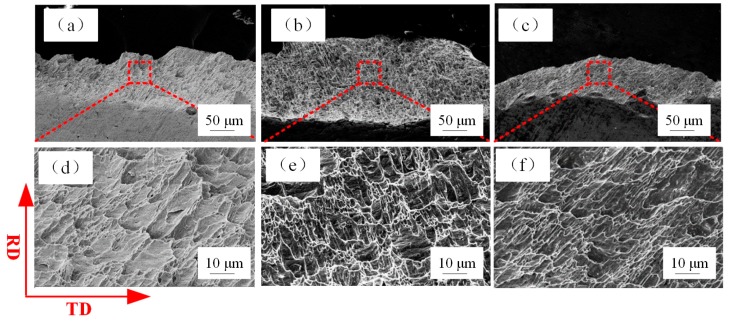
Scanning electron microscope (SEM) fractographs of sheet samples with thicknesses (**a**) 600 µm, (**b**) 400 µm, (**c**) 200 µm, where (**d**), (**e**), (**f**) are the enlarged view of the dotted rectangle in (a), (b), (c), respectively.

**Figure 6:**
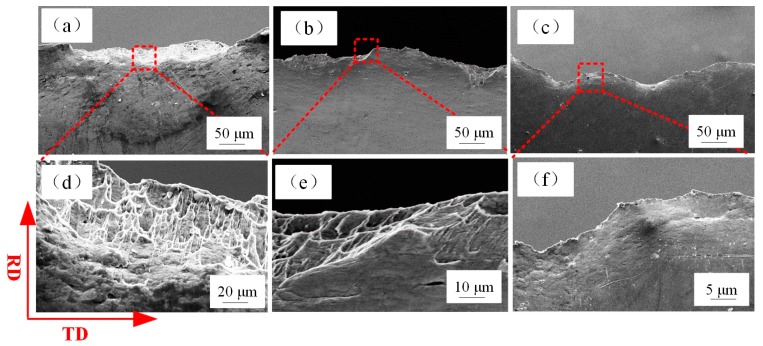
SEM fractographs of foil samples with thicknesses of (**a**) 80 µm (**b**) 40 µm, and (**c**) 20 µm, where (**d**), (**e**), (**f**) are an enlarged view of the dotted rectangle in (a), (b), (c).

Figure 4 shows the fracture angle of tensile-tested specimens of different thicknesses. The SEM fractographs of tensile-tested samples with thicknesses of 600, 400, 200 µm are shown in Figure 5. Many micro-voids were observed on the fracture surface. The increase of grain boundary fraction (as is shown in Figure 8, the decrease in grain size means an increase in grain boundary fraction in the same volume) and the concentration of stress around the grain boundary lead to grain boundary sliding and pile-up, which result in the generation of micro-voids on the fracture surface during the tensile deformation process. The SEM fractographs of tensile test samples with thicknesses of 80, 40, 20 µm are shown in Figure 6. The number of micro-voids decreased with the reduction in thickness, as shown in Figure 6a,b. In Figure 6c, no micro voids were present on the fracture surface. The changes in the fracture surfaces for t = 80, 40, and 20 µm indicate that the fracture morphology transformed from dimple pattern into typical knife edge rupture. 

**Figure 7:**
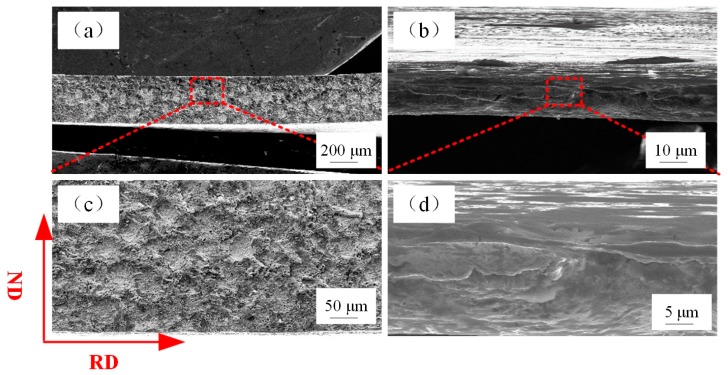
Cross-sectional photographs of fracture position with sample thickness of (**a**) 600 µm, (**b**) 20 µm, where (**c**), (**d**) are an enlarged view of the dotted rectangle in (a), (b).

Figure 7 presents the cross-sectional images of the fracture position of metal sheet (t = 600 µm) and metal foil (t = 20 µm). It is apparent that the metal foil with a thickness of 20 µm was extremely elongated and fractured during the tensile deformation process, which demonstrated ductile fracture of metal foil although the fracture stress was low. The decrease of fracture stress of the metal foils with reduction in thickness can be attributed to localized deformation during the early stretching process along the gage length. 

### 3.3. Microstructure 

**Figure 8:**
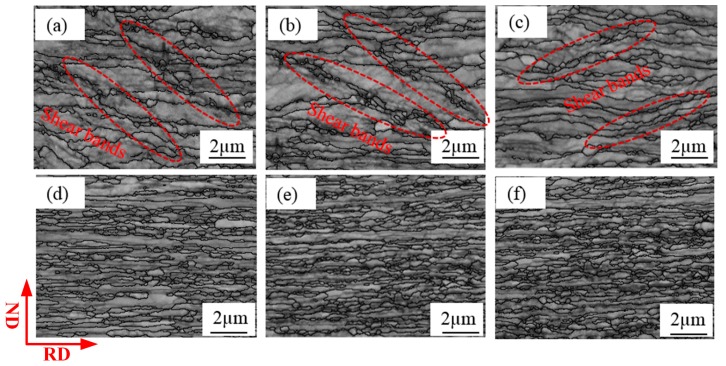
Electron backscatter diffraction (EBSD) images for the rolling specimens with thicknesses of (**a**) 600 µm, (**b**) 400 µm, (**c**) 200 µm, (**d**) 80 µm (**e**) 40 µm, (**f**) 20 µm.

The grain size and misorientation angle distribution of samples with different thicknesses can be different because of repeated rolling, which can further result in differences in mechanical properties. Figure 8 shows EBSD images of pure copper sheets and foils after rolling without intermediate annealing. After calculation, the average grain sizes are recorded in Table 2. The grain layer thinned and sub-grain structures occurred with repeated rolling. The grain size decreased from ~420 nm to ~310 nm when the thickness of Cu sheet decreased from 600 µm to 200 µm. However, it should be noted that grain refinement is insignificant when the thickness of Cu foil is rolled thinner than 80 µm. It is observed that there are many shear bands in the microstructure of Figure 8a–c and the direction of the shear band is ~45° to the rolling direction. However, grain boundary is almost parallel to rolling direction when the thickness is less than 80 µm, as is shown in Figure 8d–f, which may lead to the samples fractured ~45° to the vertical axis with thicknesses of 600, 400, 200 µm but ~90° to the vertical axis with thicknesses of 80, 40, 20 µm.

**Figure 9:**
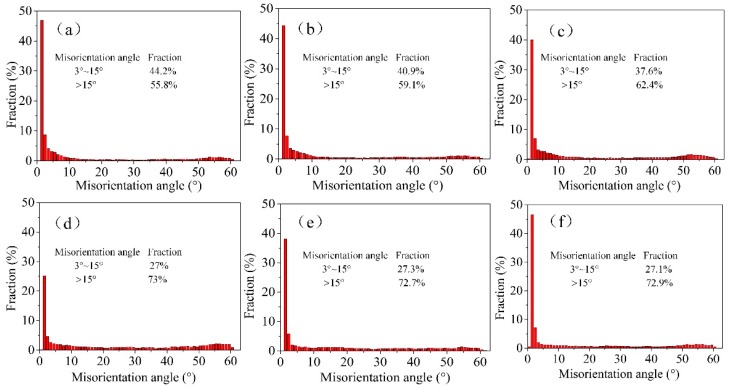
Misorientation angle distribution of rolled samples with thicknesses of (**a**) 600 µm, (**b**) 400 µm, (**c**) 200 µm, (**d**) 80 µm, (**e**) 40 µm, (**f**) 20 µm.

Figure 9 presents the misorientation angle distribution of rolled samples with different thicknesses. The flow stress is related to the contribution from both low angle boundaries (LAGBs) and high angle boundaries (HAGBs). HAGB has larger resistance to start a dislocation motion than LAGB, which means that high angle grain boundary leads to larger GB yield stress. The fraction of HAGBs increased from ~55.8% to ~73% when the thickness of Cu sheet decreased from 600 µm to 80 µm. This means the stress needed to yield GB is increased when the thickness of Cu sheet is reduced from 600 µm to 80 µm. Large HAGB fraction leads to higher fracture stress. However, there is little change in the misorientation angle distribution when the thickness of Cu foil is reduced from 80 µm to 20 µm. 

## 4. Discussion 

### 4.1. Dislocation Density

**Figure 10:**
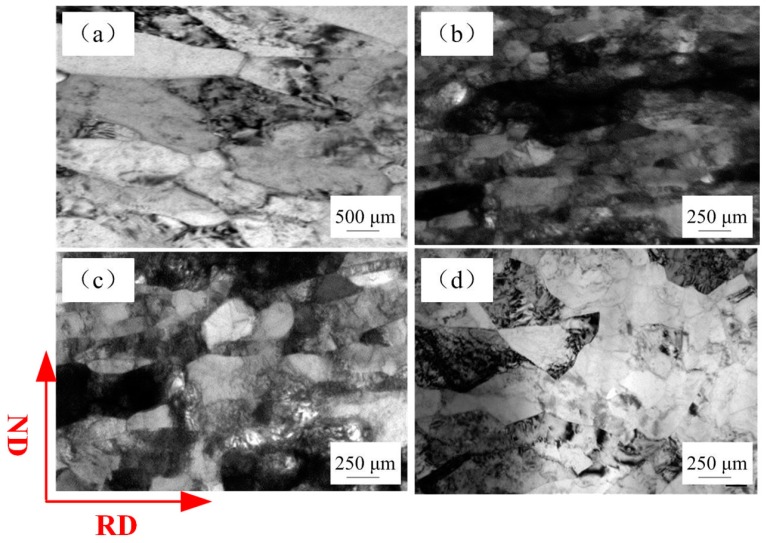
Transmission electron microscopy (TEM) images of the copper samples with different thicknesses. (**a**) 600 µm, (**b**) 80 µm, (**c**) 40 µm, (**d**) 20 µm.

The strengthening effect of polycrystalline metal is closely related to dislocations. The relationship between flow stress and the total dislocation density is given by [20]:

(1) $$\sigma_{f}\left(\varepsilon \right)=\sigma_{0}+\alpha \mathrm{bG}\sqrt{\rho }$$

where α and $\sigma_{0}$ are constants, G, b, ρ are the shear modulus, Burgers vector, and dislocation density, respectively. It appears that the strength of Cu foil decreases because of the reduction in dislocation density. Figure 10 presents the TEM images of copper samples with thicknesses of 600 µm, 80 µm, 40 µm, and 20 µm. It is observed that grain refinement is non-significant when the sample thickness is thinner than 80 µm and the grain size is maintained at ~260 nm, which is consistent with what has been observed by EBSD shown in Figure 8. 

By comparing the TEM images of Figure 10a,b, it is found that ultrafine microstructure generated and multiplicated due to the repeated rolling and ultra-high strain, and sub-grain structures and dislocation cells are easily observed when the thickness of foil is 80 µm. This leads to the increase in dislocation density and work hardening effect, and the fracture stress is increased. With further deformation, some lath boundaries became curved, which may accelerate the process of boundary sliding of pure metal deformation [21,22]. Ultra-high strain leads to severe dislocation pile-up and stress concentration at the grain boundary, such that the dislocations may penetrate through the grain boundary into the adjacent grain [23] when the concentrated stress is large enough. Therefore, the dislocation density decreases and the concentrated stress is relieved. Furthermore, ultra-thin grain layers lead to poor dislocation storage ability of grains and the dislocation density in the thickness direction is gradually saturated, which makes the unlike dislocations of adjacent grains easily move to the same grain boundary and annihilate each other [19]. This contributes to the release of the cumulative distortion energy and the tendency of softening effect. The softening effect occurs and the fracture stress is decreased.

### 4.2. Interfacial Energy

The fracture stress of Cu sheet increased with the decrease of thickness and can be attributed to grain boundary strengthening effect [15,24]. Fan [10] established a model that the polycrystalline materials can be divided into two phases: the phases of grain interior and grain boundary network. The fracture stress can be given by:

(2) $$\sigma_{c}=f_{\mathrm{gb}}\sigma_{\mathrm{gb}}+f_{i}\sigma_{i}$$

where σ_gb_ and σ_i_ are the fracture stresses of grain boundary and interior, respectively, and f_gb_ and f_i_ are the corresponding volume fraction of grain boundary and interior, respectively. Soifer et al. [25] found that grain boundaries in annealed pure Cu were 1.5 times as hard as grain interior. Therefore, the grain interior has lower fracture stress than the grain boundary. The strength of material increases with decreasing grain size, which is due to increase in the fraction of grain boundary and grain boundary strengthening effect. 

When the thickness of the material is reduced to less than 80 µm, there is no significant change in grain size, but the strength of the material is gradually reduced, so the Fan’s model cannot be applied. In the nanocrystalline metal foils, the interfacial excess volume and interfacial energy decreases with thinning grain layer [26], which leads to the decrease in resistance to dislocation motion and strengthening effect resulting from grain boundaries. Furthermore, “thinner is weaker” results from the competition between the strengthening effect of interior grain boundary and the weakening effect of exterior surface [27]. Accordingly, a surface layer model [28] (SLM) was proposed to explain the sample size effect. This model is based on the grain boundary strengthening and surface grain effects. The fracture stress of the material consists of two types: the flow stress of inner grains and surface grains. Based on this model, the flow stress of material can be expressed as below:

(3) $$\sigma_{c}=f_{s}\sigma_{s}+f_{\mathrm{in}}\sigma_{\mathrm{in}}$$

where σ_s_ and σ_in_ are the flow stresses of the surface grains and internal grains, respectively, and f_s_ and f_in_ are the corresponding volume fractions of the surface grains and internal grains, respectively. The surface grains have less constraint than the inner grains; the dislocations are almost distributed near the triple junction in the surface layer but are equally distributed inside the grain in the interior region [29]. Therefore, the grain rotation and grain boundary sliding are more likely to occur on the surface grains, which leads to lower flow stress of the surface grains. More surface grains result in smaller fracture stress. Surface weakening effect is increasingly significant for mechanical properties with reduction in thickness, which contributes to the softening effect.

### 4.3. Hall-Petch relationship

**Figure 11:**
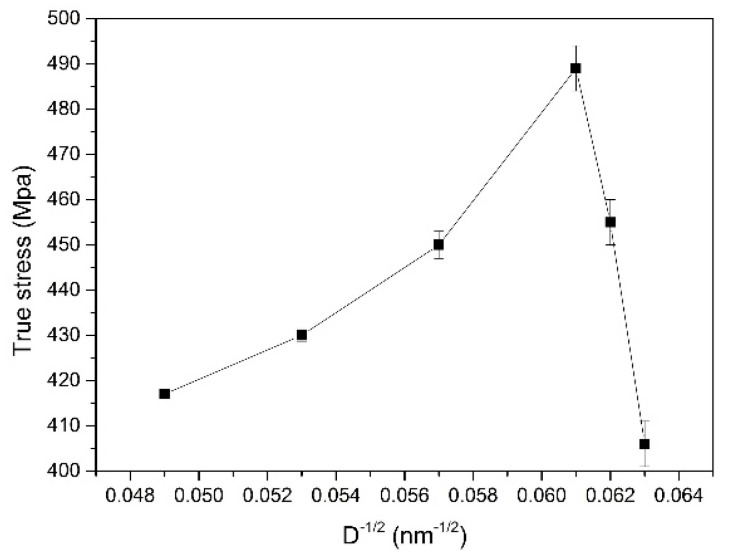
The Hall-Petch relationship in micro-rolling with different grain sizes.

For deformed material, the flow stress is related to the grain size by the classical Hall-Petch relationship [15,24]:

(4) $$\sigma \;\left(\varepsilon \right)=\sigma_{0}\left(\varepsilon \right)+k_{1}\left(\varepsilon \right)D_{GB}^{-1/2}$$

where σ (ε) and $D_{\mathrm{GB}}$ are the flow stress at a particular strain and boundary spacing, respectively. $\sigma_{0}\left(\varepsilon \right)$ and $k_{1}\left(\varepsilon \right)$ are material constants at a given strain. Figure 11 presents the Hall-Petch relationship in micro-rolling with different grain size. It is obvious that the Hall-Petch relation is valid when D^1/2^ < 0.06 nm^1/2^ (D > ~270 nm). The flow stress increases as the grain size decreases and is attributed to the strengthening effect of grain boundary and the accumulation of dislocations. However, an inverse Hall-Petch relationship [30,31] can be observed when D^1/2^ > 0.06 nm^1/2^ (D < ~270 nm). When the sample thickness is less than 80 µm, the fracture stress decreases from 489 MPa to 406 MPa with the insignificant refinement of grain size.

**Figure 12:**
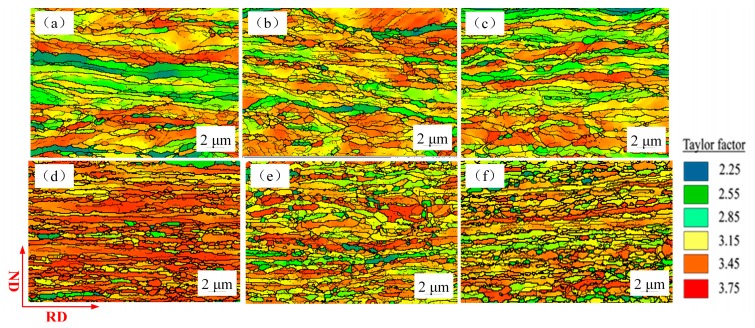
Distribution of Taylor factor of the samples with the thicknesses of (**a**) 600 µm, (**b**) 400 µm, (**c**) 200 µm, (**d**) 80 µm, (**e**) 40 µm, (**f**) 20 µm.

The grain boundary yield stress is closely related to its energetic state and structure. At ultra-high strain, it is found that many LAGBs evolve into HAGBs because of the repeated rolling (as shown in Figure 9). The high fraction of HAGBs suggests that grain boundary sliding and grain rotation may be important deformation mechanisms during micro-rolling of pure Cu foil. $\sigma_{0}\left(\varepsilon \right)$ is related to the dislocation density which can be approximated by 1.5 S_V_ θ/b, where S_V_ is the area of boundaries per unit volume and θ is the misorientation angle. Equation (5) can be given by [15]:

(5) $$\sigma_{f}\;=\sigma_{0}+M\alpha G\sqrt{1.5b{\left(S_{V}\theta \right)}_{\mathrm{LAGB}}}+k_{1}\left(\varepsilon \right)D_{\mathrm{HAGB}}^{-1/2}$$

where σ_0_ and α are constants, *M* is Taylor factor. Taylor factor is widely used to analyze the deformation behavior and predict the development of grain orientation and the texture of polycrystalline metals during plastic deformation [32]. Figure 12 presents the Taylor factor distribution of rolled samples with different thicknesses. Taylor factor distribution marked with different colors represent different grain orientations, which classifies the grains into “hard” and “soft” ones. The red area in Taylor factor distribution with Taylor factor value of 3.75 represents the hard grain orientation, the blue area with Taylor factor value of 2.25 represents the soft grain orientation. Higher Taylor factor value means more slip is required and more plastic work is expended in the deformation process [33]. 

The interfacial excess volume and interfacial energy of HAGB is larger than LAGB [26], which leads to larger resistance to dislocation motion of HAGB. Table 2 shows that the fraction of HAGBs of pure Cu foil (t = 80 µm) is 73%, which is larger than that of 55.8% for pure Cu sheet (t = 600 µm). This means the stress needed to yield GB is increased when the thickness of Cu sheet is reduced from 600 µm to 80 µm. The large fraction of HAGBs leads to higher fracture stress. The $\theta_{LAGB}$ is increased from 5.4° (t = 600 µm) to 6.9° (t = 80 µm) but then decreased to 5.7° (t = 20 µm). HAGB has larger resistance to start a dislocation motion than LAGB, which means that high angle grain boundary leads to larger GB yield stress. Therefore, the softening effect can be attributed to the decreasing value of $\theta_{LAGB}$ when the sample thickness is less than 80 µm.

The values of *M* and $\theta_{LAGB}$ with different samples recorded in Table 2 explain the variation in fracture stress with decrease in thickness. In micro-scale plastic deformation, the traditional Hall-Petch relation is not applicable for the flow stress of deformed copper foils. The strength contribution should be introduced as a variable parameter which is determined by the misorientation angle and Taylor factor.

## 5. Conclusions

The pure copper strips with different thicknesses in the range of 20 µm to 600 µm were rolled by adopting the asynchronous foil rolling technology through independently developed 3M mill. Combined with the mechanical property tests, microstructure observations, and fracture morphology, the phenomenon of apparent size effect was observed. The reasons for this phenomenon were analyzed and Hall-Petch relationship considering the distribution of misorientation angle and Taylor factor was established. The following findings are the conclusions:

(1) Apparent softening effect was observed when pure cooper strips were rolled thinner than 80 µm. As the rolling reduction was further increased, the fracture stress decreased from 489 MPa to 406 MPa when the sample thickness reduced from 80 µm to 20 µm, which is different from the traditional phenomenon.
(2) In the case of uniaxial tension test, the samples with thicknesses of 600 µm, 400 µm, and 200 µm fractured ~45° to the vertical axis. However, the fracture direction was ~90° to the vertical axis when the thickness of pure copper decreased to 80 µm, 40 µm, and 20 µm. The micro-voids on the fracture surface were decreased and the fracture type was transformed from dimple pattern into the typical knife edge rupture with reduction in thickness.
(3) By analyzing the size effect of pure copper, the decreased fracture stress with reduction in thickness is attributed to reduced interfacial energy, which resulted from the decreased interfacial excess volume and surface weakening effect. The dislocations of adjacent grains annihilate each other at the grain boundary when the thickness is less than 80 µm contributing to the release of cumulative distortion energy and tendency of softening effect. Furthermore, the influence of misorientation angle and Taylor factor also had a significant impact on the deformation behavior of pure copper strips at lower thicknesses.
(4) Modified Hall-Petch relation considering the distribution of misorientation angle and Taylor factor was established, which can be used to better clarify the relationship between the flow stress and grain size of deformed pure copper strips.

## Figures and Tables

**Table 1:** Chemical composition of pure copper (wt %).

Cu	Ag	Fe	Sb	Pb	As	S	Bi
99.40	0.5	0.005	0.002	0.005	0.002	0.005	0.001

**Table 2:** Data for rolled pure Cu without intermediate annealing.

Thickness (µm)	600	400	200	80	40	20
Grain size (nm)	~420	~356	~310	~273	~258	~250
Fraction of HAGBs (%)	55.8	59.1	62.4	73	72.7	72.9
Fraction of LAGBs (%)	44.2	40.9	37.6	27	27.3	27.1
$\theta_{LAGB}$ (°)	5.4	5.8	6.1	6.9	6	5.7
Taylor factor	3.13	3.18	3.2	3.48	3.28	3.16
